# Patterns and Limitations of Urban Human Mobility Resilience under the Influence of Multiple Types of Natural Disaster

**DOI:** 10.1371/journal.pone.0147299

**Published:** 2016-01-28

**Authors:** Qi Wang, John E. Taylor

**Affiliations:** 1 Radcliffe Institute for Advanced Study, Harvard University, Cambridge, Massachusetts, 02138, United States of America; 2 Civil Engineering Network Dynamics Lab, Charles E. Via, Jr. Department of Civil and Environmental Engineering, Virginia Tech, Blacksburg, Virginia, 24061, United States of America; IFIMAR, UNMdP-CONICET, ARGENTINA

## Abstract

Natural disasters pose serious threats to large urban areas, therefore understanding and predicting human movements is critical for evaluating a population’s vulnerability and resilience and developing plans for disaster evacuation, response and relief. However, only limited research has been conducted into the effect of natural disasters on human mobility. This study examines how natural disasters influence human mobility patterns in urban populations using individuals’ movement data collected from Twitter. We selected fifteen destructive cases across five types of natural disaster and analyzed the human movement data before, during, and after each event, comparing the perturbed and steady state movement data. The results suggest that the power-law can describe human mobility in most cases and that human mobility patterns observed in steady states are often correlated with those in perturbed states, highlighting their inherent resilience. However, the quantitative analysis shows that this resilience has its limits and can fail in more powerful natural disasters. The findings from this study will deepen our understanding of the interaction between urban dwellers and civil infrastructure, improve our ability to predict human movement patterns during natural disasters, and facilitate contingency planning by policymakers.

## Introduction

Natural disasters have a severe impact on human societies. A recent report from United Nations International Strategy for Disaster Reduction [1] revealed that natural disasters caused 1.2 million deaths, affected 2.9 billion people, and resulted in a total of US $1.7 trillion of economic loss globally from 2000 to 2012. This situation is not expected to improve; climate change is predicted to cause more frequent natural disasters for the foreseeable future [2]. Governments and communities have developed a range of mechanisms to cope with natural disasters, among which disaster response and evacuation plans are important components. These plans evaluate the potential risks posed by different types of disasters and attempt to control and minimize the consequences. However, the effectiveness of these top-down plans is often questioned, because they may lack sufficient understanding and do not take into account real-world human behaviors [3–5]. Prior to Hurricane Sandy making landfall in New Jersey in October 2012, although 71 percent of the people living in evacuation areas were aware of a mandatory order to move inland, more than 50 percent failed to do so [6]. Regrettably, most of the fatalities occurred in these evacuation areas [7]. Even those who evacuated were not entirely safe: data from the US Federal Emergency Management Agency (FEMA) revealed that the flooded areas were actually much larger than the designated evacuation areas and several people who stayed in the assumed safe areas also lost their lives [8]. In the aftermath of the hurricane, New York City updated its mandatory evacuation zones based on this experience. A similar situation occurred in the Philippines a year later when Typhoon Haiyan struck the city of Tacloban. Although the government ordered city residents to evacuate and seek shelter prior to the typhoon’s arrival, instead of moving to higher ground, many took refuge in concrete buildings that were unable to withstand the strength of the wind and the accompanying floodwaters and many lives were lost when the buildings collapsed [9]. Tragedies such as these highlight the importance of developing a better understanding of how people actually react, especially in terms of their mobility and evacuation behaviors, during natural disasters and extreme events.

Human mobility plays a critical role in disaster response and evacuation strategies. First, and possibly most importantly, it determines the effectiveness of evacuation efforts. As Pan et al. [10] pointed out, overcrowding and crushing during emergency situations can cause incidents and thus injuries and the unnecessary loss of lives. Alarmed by the approach of a severe snow storm in December 2013, the U.S. city of Atlanta, Georgia, issued a snow storm warning and advised people to leave school and work early and return home. The unfortunate consequence of this warning was that residents all crammed onto the city’s roads and highways at the same time, causing a city-wide traffic jam. Many were still stuck on the roads when the storm hit, forcing them to abandon their vehicles and seek shelter [11]. Without a deeper understanding of human movements during natural disasters, the same situation is bound to occur again. Second, human mobility also has an impact on the effectiveness of information communications during emergencies. When a region’s communications infrastructure is damaged by a natural disaster, human mobility effectively determines the bandwidth of emergency information networks and thus the speed and width of information diffusion [12]. In this situation, peer-to-peer connections can create ‘Mobile ad-hoc networks’ (MANETs) or ‘Pocket switched networks’ (PSNs) using mobile communication devices such as cell phones [13, 14]. These temporary networks can provide critical information about potential dangers and/or evacuation routes and hence reduce injuries, fatalities, and economic loss [15]. Third, accurate human mobility predictions can also potentially save lives. In both Hurricane Sandy and Typhoon Haiyan, if it had been possible to identify vulnerable individuals inside the flooding zones and areas that experienced infrastructure damages and provide them with detailed instructions, some lives might have been spared. The critical roles of human mobility related to all three of these aspects call for in-depth investigations to build our understanding of how best to work with real-world human behaviors in disaster situations.

Despite its importance, little research into human mobility has been reported related to mobility under the influence of natural disasters, i.e. in perturbed states. While several fundamental characteristics of generic human mobility have been identified, research on perturbed human mobility is fragmented and little has been done to discover fundamental patterns. Also, these studies mostly focus on one case or one type of natural disaster. It is therefore unclear whether the findings of these studies can be extended to other extreme events. To address this research gap, this study attempts to take an initial step towards identifying patterns in perturbed human mobility by examining human mobility under the influence of multiple types of natural disasters. This study is based on a large quantity of human movement data collected from Twitter. This data collection effort has taken almost two years, providing us with human mobility data for a number of different natural disasters around the world, including tropical cyclones (hurricanes and typhoons), winter storms, wildfires, earthquakes, and severe rainstorms. By analyzing and comparing the data from these events, we attempted to uncover universal patterns in the movements of a perturbed urban population.

The paper is organized as follows. After reviewing related work about human mobility and how it could potentially be influenced by natural disasters, we propose three hypotheses. This is followed by sections introducing our data collection and analytical methods. Then, the results, the findings and their implications are discussed. The paper concludes by addressing the study’s limitations and presenting our conclusions.

## Background

There has been a great deal of research into general human mobility patterns. Using both currency circulation data and mobile phone data, researchers have confirmed that human movements follow a power-law distribution, with *β* values ranging from 1.59 to 1.88 [16–19]. This means that human movements can generally be described by the Lévy flight model, which is typically found in animals’ movement patterns [20]. Research has also shown that individual movement trajectories exhibited similar shapes after being rescaled by the radius of gyration [17]. Song et al. [21] investigated a large dataset containing a year’s worth of location information for 1 million mobile phone users and observed three unique characteristics of human mobility which both the Lévy flight model [16] and the continuous-time random-walk model [22, 23] failed to explain. These characteristics were: (1) a decreasing tendency for a person to visit new locations; (2) an uneven visitation frequency for different locations; and (3) an ultraslow diffusion, which meant people tended to return to the same locations (e.g. home, office, etc.). Based on these observations, Song et al. [21] developed a new individual-mobility model that incorporated two unique generic mechanisms: exploration and preferential return. However, although this new model is more representative of human mobility patterns than previous models, it still only captures long-term spatial and temporal scaling patterns.

Human movement at the city scale has also been investigated. At this level, periodic modulations characterize human mobility [21]. Noulas et al. [24] studied human mobility in 31 large cities around the world, and found that the global movements followed a power-law distribution. They also found human mobility in all the cities studied followed almost the same pattern. Perhaps unsurprisingly, several studies have also found that people exhibit characteristics of periodicity governed by 24 hour and 7 day temporal cycles in returning to primary locations [25, 26]. Human movements have also been shown to follow highly efficient trajectory configurations during their daily movements. Schneider et al. [27] reported that people are highly efficient when performing their daily trips, following only 17 trajectory configurations out of over a million possible trajectories. These patterns of human mobility observed in urban areas enabled the possibility to predict and simulate human movements in an urban environment [28, 29]. Additionally, this research inspired other studies to understand long-term impacts on urban spatial interactions and transportation infrastructure [30, 31]. Toole et al. [32] studied the coupling phenomenon between human mobility and social ties and demonstrated that an individual’s social network is correlated with mobility behavior. Such a finding is not only important to understand human mobility in steady states, but also can play a key role in predicting human mobility in disasters and emergencies. As Sampson [33] pointed out, social infrastructure is a vital element to reduce damage and save lives during disasters.

While these human mobility studies have improved our knowledge about general mobility patterns, it seems likely that a change in the environment, particularly a major event such as a natural disaster, will significantly perturb these routine patterns. Bagrow et al. [34] used mobile phone billing data to track people’s communication in 8 emergency events (including bombings and earthquakes) and 8 non-emergency events (such as sports events and concerts). Their results showed that the emergency information tends to diffuse globally while the non-emergency information is more spatially constrained. Horanont et al. [35] studied the relationship between weather conditions and people’s everyday activities and discovered that some types of weather conditions can significantly influence human movements, although the level of influence on individuals varied greatly. These findings indicate that unusual events and changes in the natural environment can indeed influence people’s activities. Natural disasters can cause major population migration. Morrow-Jones and Morrow-Jones [36] studied an 8-year dataset and confirmed that natural disasters can cause involuntary migration. This can occur on a large scale; Bengtsson et al. [37] tracked population movements in Haiti using cell phone data and found that earthquake and disease infection caused as much as 20% of the population to leave the capital city, Port-au-Prince. Several studies have also examined the reciprocal influence between human mobility and epidemics[38, 39].

While these studies demonstrate that human movement trajectories during disasters do deviate from their normal steady states, research in this area is fragmented and not enough effort has been devoted to discovering fundamental patterns in human mobility under the influences of natural disasters. Many factors have constrained more extensive and in-depth research, but a key issue is the inherent unpredictability of natural disasters. Current technology is still ineffective in predicting the occurrence of natural disasters such as earthquakes and tornadoes, and even though we now have some advance warning of some types of natural disasters, particularly tropical cyclones, winter storms, and rainstorms, researchers and practitioners are still unable to accurately forecast their precise paths, strength, and influence. Hurricane Sandy had already caused extensive damage in Jamaica and Cuba before it arrived in the U.S. several days later, but the nation still suffered 73 deaths and about $65 billion of economic loss [40]. The failure of one of the world’s most developed countries to minimize the damage from a significant impending natural disaster when the devastation it had already wreaked in two other countries had been featuring on the evening news for days highlights the challenges involved in protecting urban dwellers from natural disasters. This unpredictability also makes it difficult to collect empirical human movement data from multiple types of natural disasters, and thus researchers have limited data at their disposal when seeking to examine the fundamental attributes of perturbed human mobility.

One of these fundamental attributes is resilience. Understanding and quantifying the resilience of human mobility could provide a key indicator for measuring the vulnerability and adaptability of human society when facing natural disasters [41–43]. It could help predict human movements in urban areas and shed new light on the interdependence between human mobility and civil infrastructure, providing invaluable knowledge that will help define the shape of the decision-making landscape for socio-ecological systems [41]. However, there has been only limited research into understanding and quantifying the resilience of human mobility. A recent examined human movement under the influence of Hurricane Sandy and discovered that human mobility does indeed possess inherent resilience [44]. The study’s findings revealed that the power-law still described New Yorkers’ movements during Hurricane Sandy and that the values for the center of movement and the radius of gyration were correlated with their values during a steady state. This correlation suggests the possibility of predicting the pattern of perturbed human mobility. Nevertheless, the study did not examine whether this resilience can withstand the pressures of other types of disasters or disasters with more extensive impacts and damages. There is a critical gap in research on human mobility perturbation and resilience under the influence of multiple types of natural disasters. Such research is critical for predicting human movements during natural disasters and exploring the interdependence between human mobility and civil infrastructure. Ultimately, a better understanding of human mobility in highly stressful disaster situations will promote public safety by identifying the most effective ways to predict human locations and travel patterns, thus facilitating the protection of vulnerable individuals from potential harm and injury.

## Hypotheses Development

Based on the results from previous studies, we posited several hypotheses to examine human mobility resilience. Resilience in human mobility refers to the ability of human movement to absorb shocks, maintain its fundamental attributes, and return to its steady state equilibrium in response to natural disasters [44]. The hypotheses were then tested for each natural disaster case. As mentioned earlier, human mobility can be described by a power-law [16, 17] and although extreme weather can significantly influence human movements [34, 35], this power-law holds even during a strong hurricane [44]. Therefore, we propose our first hypothesis:

**Hypothesis 1:** A power law governs human urban travels in multiple types of natural disaster.

Researchers have found that the center of mass of human movements and the radius of gyration can fundamentally describe individual human movements [17]. Moreover, researchers have found the values of these two factors during the perturbation state to be correlated to their values during the steady state [44]; people tend to seek refuge in areas that are already familiar to them. We therefore seek to examine whether this can be extrapolated to multiple types of natural disasters, and proposed the following two hypotheses:

**Hypothesis 2:** Shifts in the distances of centers of movement during natural disasters are positively correlated with the values of the radius of gyration in steady states.

**Hypothesis 3:** Values of radius of gyration during natural disasters are positively correlated with the ones in steady states.

## Data Collection

Much empirically grounded human mobility research utilizes mobile phones to track human mobility [17, 21, 27, 39, 45]. The data precision of these studies is limited to the coverage area of each mobile phone tower, which is typically around 1~3km^2^. While such precision has been instrumental in developing an understanding of general patterns of human mobility over larger scales (e.g., a state or a country), it may lack the necessary precision to capture mobility changes caused by disasters and other extreme events that unfold at smaller scales (e.g., a city).

To overcome this limitation, Twitter was used to collect high-resolution human mobility data in this study. Twitter is an online social networking media that allows people to post statuses that are limited to 140 characters, called tweets. It has over 645 million active users [46] and they post about 500 million tweets per day [47]. Users can enable a function which automatically adds location information, called a geotag, to each tweet they post. Each geotag contains the geographical coordinate at which the tweet was posted. Numerous studies have utilized the platform to study communication and geo-social networking [43, 48]. Using the Twitter public API, we developed a method to collect geotagged tweets around the world. Refer to [49] for details about the data collection system.

Human mobility data before, during and after fifteen natural disaster events from five types of natural disasters were collected to conduct this study. We collected the data over a two-year period. Then we reviewed natural disasters that occurred during the period and retrieved human mobility data from the affected urban areas. We identified fifteen disasters that provided sufficient data for analysis. These fifteen disasters divide into the following groups: four typhoons, three severe winter storms, three earthquakes, two wild fires, and three record-breaking rainstorms. They included a total of 3,673,626 geo-tagged tweets from 212,735 individuals. A summary of these events and data can be found in Table 1.

**Table 1:** Summary of Natural Disasters and Collected Data.

Type	Name	Location	No. of Tweets	No. of Users
Typhoon	Wipha (Tokyo)	Tokyo, Japan	849,173	73,451
	Halong (Okinawa)	Okinawa, Japan	166,325	5,124
	Kalmaegi (Calasiao)	Calasiao, Philippines	21,698	1,063
	Rammasun (Manila)	Manila, Philippines	408,760	27,753
Earthquake	Bohol (Bohol)	Bohol, Philippines	114,606	7,942
	Iquique (Iquique)	Iquique, Chile	15,297	1,470
	Napa (Napa)	Napa, USA	38,019	1,850
Winter storm	Xaver (Norfolk)	Norfolk, Britain	115,018	8,498
	Xaver (Hamburg)	Hamburg, Germany	15,054	2,745
	Storm (Atlanta)	Atlanta, USA	157,179	15,783
Thunderstorm	Storm (Phoenix)	Phoenix, USA	579,735	23,132
	Storm (Detroit)	Detroit, USA	765,353	15,949
	Storm (Baltimore)	Baltimore, USA	328,881	14,582
Wildfire	New South Wales^a^ (1)	New South Wales, Australia (1)	64,371	9,246
	New South Wales^a^ (2)	New South Wales, Australia (2)	34,157	4,147

^a^The wildfire covered 290,000 acres, and we picked the two most severe areas that were close to urban areas.

## Data Analysis and Results

To explore the proposed hypotheses, we conducted multiple analyses on the human mobility data collected from Twitter. To test the first hypothesis and determine whether natural disasters changed the fundamental power law that describes human mobility, we first calculated the displacements. As described in detail previously [50], a displacement is the distance over two consecutive points from one individual. It was calculated using the Haversine formula [51]:

$$d=2r\times {\text{sin}}^{-1}\left(\sqrt{{\text{sin}}^{2}\left(\frac{\phi_{2}-\phi_{1}}{2}\right)+\text{cos}\phi_{1}\phi_{2}\;{\text{sin}}^{2}\left(\frac{\varphi_{2}-\varphi_{1}}{2}\right)}\right)$$

Where *r* is the earth radius, which approximately equals to 6,367,000 meters, *ϕ* is the latitude, and *φ* is the longitude

We fitted the displacement data into truncated power-law distribution [17, 52, 53]. The truncated power-law distribution can be captured by the following equation:

$$P(\Delta r)\propto \Delta r^{-\beta }e^{-\lambda \Delta r}$$

Where Δ*r* is the displacement, *β* is the scaling parameter, and *λ* is the exponential cutoff value.

To test the goodness of fit, we conducted the Maximum Likelihood Estimation test to compare the truncated power-law distribution to both the exponential distribution and lognormal distribution. Maximum Likelihood Estimation is a statistical method to estimate which statistical model has higher goodness of fit to the empirical data [4, 54, 55]. The Python package *powerlaw* was used for fitting and Maximum Likelihood Estimation. Refer to [50] for more details about the data analysis.

**Fig 1:**
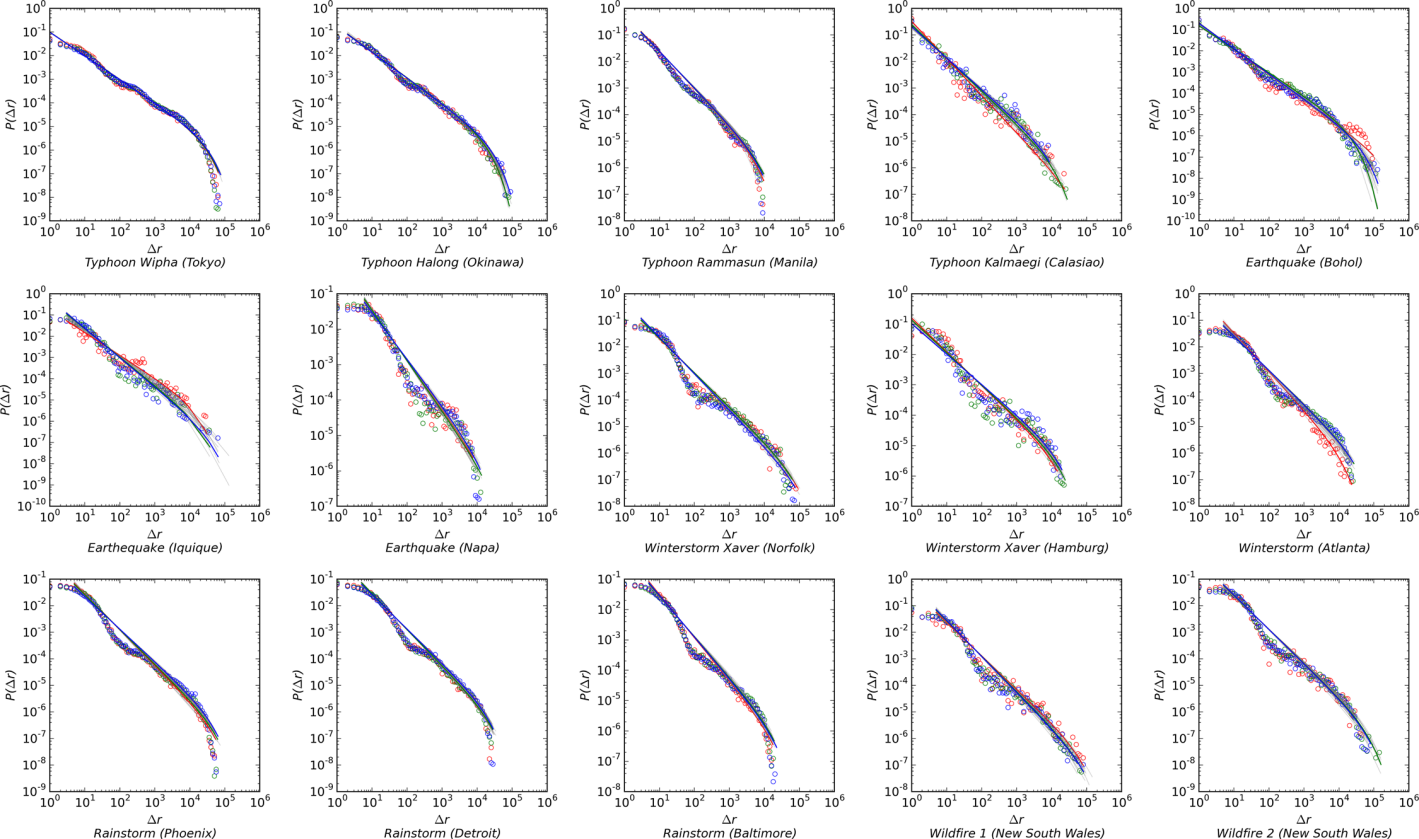
Human Movement Data Fitting Results.

The results of the fitting are shown in Fig 1. The red circles represent the movements during the 24-hour period when the disaster occurred. The green circles represent the movements before the disasters occurred, while the blue circles represent the aftermath. The red, green and blue lines are the fitting results. The Maximum Likelihood Estimation (MLE) test results are provided in S1 Table. The results suggest that the power-law still dominates human mobility in most cases across different types of disasters. In all cases, the MLE tests demonstrated that each daily displacement data fits with the power-law distribution better than the exponential distribution (*p*-value<0.001). Out of 471 days, we found that 455 days followed the power-law distribution better than the lognormal distribution. Therefore, 96.6% of the days in our sample follow the power law distribution providing support for Hypothesis 1.

To test Hypothesis 2 and Hypothesis 3, we analyzed the shifting distances of the centers of human movement trajectories during the natural disasters in each affected city. We also documented the changes of the radius of gyrations of human movements from the steady states to the perturbation states. We first calculated each individual’s center of mass of movements from both perturbation states ${\vec{r}}_{CM}^{P}$ and steady states ${\vec{r}}_{CM}^{S}$ using the following equation:

$${\vec{r}}_{CM}=\frac{1}{n(t)}\sum_{i=1}^{n(t)}{\vec{r}}_{i}$$

Where ${\vec{r}}_{i}$ was a coordinate, and *n(t)* was the number of places one visited within the 24-hour period. The perturbation state was set as the 72-hour period when a natural disaster occurred, and the steady state was set as another 72-hour period which occurred two weeks before the perturbation state. Using the center of mass in the steady state and in the perturbation state, the shifting distance *Δd*_*CM*_ was calculated using the following equation:

$$\Delta d_{CM}=|{\vec{r}}_{CM}^{P}-{\vec{r}}_{CM}^{N}|$$

We also calculated the radius of gyration in both the perturbation states *r*_*g*_^*P*^ and the steady states *r*_*g*_^*N*^ using the following equation:

$$r_{g}=\sqrt{\frac{1}{n}\sum_{t=1}^{n}{\left[2r\times {\text{sin}}^{-1}\left(\sqrt{{\text{sin}}^{2}\left(\frac{\phi_{t}-\phi_{c}}{2}\right)+\text{cos}\phi_{1}\;\text{cos}\phi_{t}\;{\text{sin}}^{2}\left(\frac{\varphi_{t}-\varphi_{c}}{2}\right)}\right)\right]}^{2}}$$

Where *n* is the total number of visited locations from one individual, *t* is each location, *ϕ* is the latitude, and *φ* is the longitude, *c* is the center of mass of movements. The states were the same periods we used to calculate the center of mass.

Analytic results demonstrated that *Δd*_*CM*_ are strongly correlated with *r*_*g*_^*S*^ in thirteen out of the fifteen cases. The two outlier cases were particularly extreme and/or unusual events, which is discussed later in the next section. The correlation coefficients and *p*-values are presented in Table 2. We found Hypothesis 2 to be supported in thirteen cases. We also tested the correlation between *r*_*g*_^*P*^ and *r*_*g*_^*S*^. The correlation coefficients were positive and significantly different from zero in eleven out of fifteen cases, as shown in Table 3. Therefore, Hypothesis 3 was supported in eleven cases. Again, the outlier natural disaster cases are discussed further in the next section.

**Table 2:** Correlation between *Δd*_*CM*_ and *rgS*.

Type	Name/Location	Correlation Coefficient
**Typhoon**	Wipha (Tokyo)	0.539672692***
	Halong (Okinawa)	0.399066158***
	Kalmaegi (Calasiao)	0.412774447*
	Rammasun (Manila)	0.57926189***
**Earthquake**	Bohol (Bohol)	0.270450514***
	Iquique (Iquique)	0.15591484
	Napa (Napa)	0.463082337***
**Winter storm**	Xaver (Norfolk)	0.555180382***
	Xaver (Hamburg)	0.257535062
	Storm (Atlanta)	0.678411423***
**Rainstorm**	Storm (Phoenix)	0.533596975***
	Storm (Detroit)	0.452612074***
	Storm (Baltimore)	0.413542843***
**Wildfire**	New South Wales (1)	0.896617706***
	New South Wales (2)	0.292383392*

*significant at *p*< 0.05

** significant at *p*<0.01

***significant at *p*<0.001

**Table 3:** Correlation between *rgP* and *rgS*.

Type	Name/Location	Correlation Coefficient
**Typhoon**	Wipha (Tokyo)	0.524405***
	Halong (Okinawa)	0.215695***
	Kalmaegi (Calasiao)	0.149604
	Rammasun (Manila)	0.288469***
**Earthquake**	Bohol (Bohol)	0.031066
	Iquique (Iquique)	-0.08923
	Napa (Napa)	0.33824*
**Winter storm**	Xaver (Norfolk)	0.332673***
	Xaver (Hamburg)	0.198529
	Storm (Atlanta)	0.250897***
**Rainstorm**	Storm (Phoenix)	0.341179***
	Storm (Detroit)	0.245938***
	Storm (Baltimore)	0.243112***
**Wildfire**	New South Wales (1)	0.799249***
	New South Wales (2)	0.507008***

*significant at *p*< 0.05

** significant at *p*<0.01

***significant at *p*<0.001

## Discussion

Our results, in aggregate, demonstrate that natural disasters do indeed influence human movements in urban areas, although the impact can vary in terms of severity and duration. In this section we discuss the results of each of our hypotheses in relation to previous research and its implications for future research and practice. These findings add to our understanding of the perturbation and resilience of human mobility during natural disasters.

In our first hypothesis, we assumed that natural disasters would not fundamentally change human mobility patterns and that the power-law distribution would continue to describe human movements. Results from data fitting and Maximum Likelihood Estimation tests [52] show that although perturbed by different types of natural disaster, human movements in almost all the cases and over 95% of the days we collected data were still governed by the power-law. This finding aligns with previous studies that showed general human mobility follows a power-law distribution, with beta values ranging from 1.59 to 1.88 [16–19]. The differences between the calculated *β* values here and the values reported in other studies may be due to the fact that the values in our study were derived from higher precision location data in more tightly constrained geographical areas.

While previous research has demonstrated that human mobility follows similar distribution in large population centers [24], our finding reveals that human mobility possesses an even more universal pattern. We discovered that power-law governs human movements in perturbation states impacted by natural disasters. Also, while we included some smaller cities and less urbanized areas, truncated power-law still dominates the distribution of human mobility regardless the urban setting and population density.

In our second hypothesis, we assumed that the values of the shifts in the distances of the centers of individual movements (*Δd*_*CM*_) are correlated with the values of the radius of gyration in steady states (*r*_*g*_^*S*^). Our analysis demonstrates that while the correlation is supported in most cases, this correlation was not statistically significant in either the 2014 Iquique earthquake or the 2013 Xaver winter storm in Hamburg. Similarly, while we observed significant correlations between the values of the radius of gyration in perturbed states (*r*_*g*_^*P*^) and those in steady states (*r*_*g*_^*S*^) in eleven cases, we did not find such correlations in four cases. In addition to the same two disasters identified in the previous analysis, no statistical correlations were supported in either the 2013 Bohol earthquake or Typhoon Kalmaegi in 2014.

The findings from both Hypothesis 2 and Hypothesis 3 are critical to understand human mobility under the influence of natural disasters. While previous research has discovered that the radius of gyration can be used to capture individual human mobility patterns [17], an individual’s radius of gyration could significantly change under the influence of natural disasters and extreme events. In cases where natural disasters have a relatively mild impact, the radius of gyration can indeed be used to understand and even predict human movement patterns, as noted in a previous study [56]. However, if the impact is severe enough, human mobility patterns observed in steady states can be significantly impacted. Thus, they can no longer be used to predict human movements in their perturbed states.

The influence of natural disasters on mobility is complex. Take the 2014 Iquique earthquake and 2013 Bohol Earthquake as examples. While both caused regional human mobility to lose resilience, they have different attributes. The Iquique earthquake, with a magnitude of 8.2, was the strongest earthquake that occurred in 2014. It impacted a city of about 180,000 residents, and caused 6 casualties. The 2013 Bohol earthquake had a magnitude of 7.2. It attacked Bohol with over 1.2 million dwellers and resulted in the deaths of more than 200 people. Such complexity could be observed in the Winterstorm Xaver which impacted much of Europe. While it significantly impacted human mobility in Hamburg, Germany, Norfolk, Britain withstood its impact with high resilience. Untangling the complexity is beyond the scope of this study, but further research is needed to evaluate the diverse influences and determine the point after which human mobility resilience collapses.

## Limitations

While Twitter is widely adopted in some countries, it is less prevalent in other places. For this study a substantial amount of empirical data was collected from Tokyo, Manila, Okinawa, Norfolk, Hamburg, Napa, Atlanta, Phoenix, Detroit and Baltimore, but less data was available from places such as Iquique, where we were able to collect less than 300 displacements (the suggested value for comparing the goodness of fit between a lognormal distribution and a power-law distribution) for several days after the Iquique earthquake. The limited number of data points could potentially influence the results, resulting in a better fit for the lognormal distribution for several days. However, we were able to retrieve more than 100 displacements each day for all the cases, and overall the number is sufficient to distinguish whether a power-law distribution or an exponential distribution fitted the data better.

## Conclusion

Human mobility in urban areas is regrettably impacted by natural and man-made disasters. Existing research has reported that changes in the natural environment can cause behavioral change and temporary, or even permanent, human migrations [36, 37]. In this study we collected empirical human movement data using Twitter to discover whether human mobility is indeed perturbed by different natural disasters, and whether the human mobility patterns observed in steady states are correlated with those during natural disasters. The data were analyzed to identify and quantify human mobility perturbation from the steady state in each case.

Our findings demonstrate that: (1) human mobility patterns are unlikely to deviate from the fundamental power-law during a natural disaster; (2) human mobility in perturbed states generally shows significant correlations with those in steady states; and (3) in the event of a particularly severe natural disaster, human mobility can become more erratic and this correlation can be lost.

The results from the empirical data revealed that power-law continues to govern human mobility during a natural disaster. This result supports the findings from several studies of general human mobility patterns [16–19]. While previous research did not distinguish between human mobility patterns in perturbation states or steady states, our findings demonstrate that the power-law is still applicable even during multiple types of natural disasters, highlighting the inherent resilience and adaptability of human mobility.

The study also contributes to research into human mobility by showing that natural disasters can significantly change human mobility patterns even where the fundamental power-law still holds. An earlier study had shown that human mobility has inherent resilience; the values of the radius of gyration in perturbation states were correlated with those in steady states in New York City when Hurricane Sandy came onshore [56]. This study extends this earlier research by examining this resilience more closely across multiple types of natural disasters, demonstrating that human mobility resilience can survive a certain level of perturbation during disasters but that more powerful disasters can destroy this resilience and force urban dwellers to adopt entirely different travel patterns.

While this study provides a first attempt to examine human mobility perturbation over a range of natural disaster types, future research can build on its findings by extending this approach to additional types of natural disasters and incorporating other influential factors as independent variables that may, or may not, be correlated with the mobility patterns (for example, the differing availability of public transportation and/or types of mobility infrastructure available). Such future research will help identify the factors that contribute significantly to human mobility perturbation. This will help policy-makers and practitioners to better predict human movements and improve disaster evacuation, response, and recovery plans.

## Supporting Information

S1 Table Complete Data Fitting Results. (DOCX)
