# Supplementary material for: Patterns and Limitations of Urban Human Mobility Resilience under the Influence of Multiple Types of Natural Disaster
Source: PLoS One. 2016 Jan 28;11(1):e0147299. doi: 10.1371/journal.pone.0147299 (PMC4731215; doi:10.1371/journal.pone.0147299)
Supplement: S1 Table — (DOCX) [file pone.0147299.s001.docx]

| **Case** | **Day** | **Scaling Parameter** | **Xmin**^1^ | **Lognormal Comparison**^2^ | **Exponential Comparison**^3^ |
| --- | --- | --- | --- | --- | --- |
| Wipha | 1 | 1.00 | 1 | 522.46*** | 15890.04*** |
| Wipha | 2 | 1.00 | 1 | 540.51*** | 17419.26*** |
| Wipha | 3 | 1.00 | 1 | 602.09*** | 17523.11*** |
| Wipha | 4 | 1.00 | 1 | 573.39*** | 17315.68*** |
| Wipha | 5 | 1.00 | 1 | 493.87*** | 16332.13*** |
| Wipha | 6 | 1.00 | 1 | 625.35*** | 18314.91*** |
| Wipha | 7 | 1.00 | 1 | 596.34*** | 18740.83*** |
| Wipha | 8 | 1.00 | 1 | 463.86*** | 16025.27*** |
| Wipha | 9 | 1.00 | 1 | 464.58*** | 15316.92*** |
| Wipha | 10 | 1.00 | 1 | 494.3*** | 15364.37*** |
| Wipha | 11 | 1.00 | 1 | 448.48*** | 15511.45*** |
| Wipha | 12 | 1.00 | 1 | 455.47*** | 14414.25*** |
| Wipha | 13 | 1.00 | 1 | 394.69*** | 16635.77*** |
| Wipha | 14 | 1.00 | 1 | 405.43*** | 17240.04*** |
| Wipha | 15 | 1.00 | 1 | 537.36*** | 18700.86*** |
| Wipha | 16 | 1.00 | 1 | 676.01*** | 18692.28*** |
| Wipha | 17 | 1.00 | 1 | 646.42*** | 21682.6*** |
| Wipha | 18 | 1.00 | 1 | 440.99*** | 15117.24*** |
| Wipha | 19 | 1.00 | 1 | 431.45*** | 14248.4*** |
| Wipha | 20 | 1.00 | 1 | 491.23*** | 17291.01*** |
| Wipha | 21 | 1.00 | 1 | 671.23*** | 19332.81*** |
| Wipha | 22 | 1.00 | 1 | 655.87*** | 16304.15*** |
| Wipha | 23 | 1.00 | 1 | 460.14*** | 15364.21*** |
| Wipha | 24 | 1.00 | 1 | 496.5*** | 15778.3*** |
| Wipha | 25 | 1.00 | 1 | 609.73*** | 18214.82*** |
| Wipha | 26 | 1.00 | 1 | 589.23*** | 16092.69*** |
| Wipha | 27 | 1.00 | 1 | 496.33*** | 17740.77*** |
| Wipha | 28 | 1.00 | 1 | 448.44*** | 18613.65*** |
| Wipha | 29 | 1.00 | 1 | 470.9*** | 15129.74*** |
| Wipha | 30 | 1.00 | 1 | 556.44*** | 15561.4*** |
| Wipha | 31 | 1.00 | 1 | 192.57*** | 14770.86*** |
| Wipha | 32 | 1.00 | 1 | 470.05*** | 16946.51*** |
| Wipha | 33 | 1.00 | 1 | 408.12*** | 15875.09*** |
| Wipha | 34 | 1.00 | 1 | 329.19*** | 16348.15*** |
| Halong | 1 | 1.08 | 2 | 56.34*** | 5505.09*** |
| Halong | 2 | 1.09 | 2 | 67.05*** | 5211.8*** |
| Halong | 3 | 1.10 | 2 | 68.03*** | 5087.1*** |
| Halong | 4 | 1.06 | 2 | 109.86*** | 4484.07*** |
| Halong | 5 | 1.11 | 2 | 83.65*** | 5229.3*** |
| Halong | 6 | 1.11 | 2 | 73.82*** | 5546.28*** |
| Halong | 7 | 1.10 | 2 | 54.37*** | 5264.73*** |
| Halong | 8 | 1.09 | 2 | 74.58*** | 4737.1*** |
| Halong | 9 | 1.14 | 2 | 69.1*** | 6005.99*** |
| Halong | 10 | 1.16 | 2 | 74.06*** | 6868.52*** |
| Halong | 11 | 1.11 | 2 | 92.59*** | 6269.46*** |
| Halong | 12 | 1.10 | 2 | 73.49*** | 5430.27*** |
| Halong | 13 | 1.10 | 2 | 105.72*** | 5376.0*** |
| Halong | 14 | 1.06 | 2 | 99.85*** | 5009.56*** |
| Halong | 15 | 1.11 | 2 | 41.38*** | 4213.7*** |
| Halong | 16 | 1.09 | 2 | 78.29*** | 5885.46*** |
| Halong | 17 | 1.10 | 2 | 68.71*** | 5547.58*** |
| Halong | 18 | 1.08 | 2 | 75.97*** | 6179.17*** |
| Halong | 19 | 1.08 | 2 | 93.99*** | 5767.33*** |
| Halong | 20 | 1.06 | 2 | 82.08*** | 5807.33*** |
| Halong | 21 | 1.08 | 2 | 126.12*** | 4984.1*** |
| Halong | 22 | 1.10 | 2 | 80.55*** | 5904.64*** |
| Halong | 23 | 1.07 | 2 | 77.8*** | 4669.98*** |
| Halong | 24 | 1.10 | 2 | 88.1*** | 5713.79*** |
| Halong | 25 | 1.07 | 2 | 69.15*** | 4866.53*** |
| Halong | 26 | 1.10 | 2 | 87.7*** | 6209.91*** |
| Halong | 27 | 1.07 | 2 | 56.76*** | 5316.41*** |
| Halong | 28 | 1.09 | 2 | 97.22*** | 6250.5*** |
| Halong | 29 | 1.10 | 2 | 51.99*** | 6206.2*** |
| Halong | 30 | 1.11 | 2 | 58.2*** | 6190.21*** |
| Halong | 31 | 1.09 | 2 | 52.82*** | 5376.95*** |
| Halong | 32 | 1.11 | 2 | 89.8*** | 6414.53*** |
| Halong | 33 | 1.08 | 2 | 64.13*** | 5415.26*** |
| Rammasun | 1 | 1.40 | 3 | 115.37*** | 12973.6*** |
| Rammasun | 2 | 1.43 | 3 | 98.08*** | 13639.57*** |
| Rammasun | 3 | 1.34 | 3 | 149.47*** | 13407.53*** |
| Rammasun | 4 | 1.35 | 3 | 157.9*** | 14362.26*** |
| Rammasun | 5 | 1.30 | 3 | 163.62*** | 12541.22*** |
| Rammasun | 6 | 1.39 | 3 | 134.25*** | 15138.92*** |
| Rammasun | 7 | 1.43 | 3 | 101.99*** | 15440.3*** |
| Rammasun | 8 | 1.34 | 3 | 139.76*** | 14925.73*** |
| Rammasun | 9 | 1.33 | 3 | 161.43*** | 13559.84*** |
| Rammasun | 10 | 1.36 | 3 | 131.48*** | 14173.97*** |
| Rammasun | 11 | 1.31 | 3 | 138.91*** | 12288.75*** |
| Rammasun | 12 | 1.32 | 3 | 163.35*** | 13792.23*** |
| Rammasun | 13 | 1.38 | 3 | 149.73*** | 16137.91*** |
| Rammasun | 14 | 1.42 | 3 | 130.32*** | 17333.32*** |
| Rammasun | 15 | 1.36 | 3 | 170.21*** | 17620.49*** |
| Rammasun | 16 | 1.41 | 3 | 191.12*** | 23310.79*** |
| Rammasun | 17 | 1.32 | 3 | 96.26*** | 11943.99*** |
| Rammasun | 18 | 1.39 | 3 | 134.38*** | 17151.55*** |
| Rammasun | 19 | 1.34 | 3 | 152.82*** | 14906.02*** |
| Rammasun | 20 | 1.38 | 3 | 145.42*** | 17952.44*** |
| Rammasun | 21 | 1.40 | 3 | 121.69*** | 17349.34*** |
| Rammasun | 22 | 1.35 | 3 | 154.43*** | 14653.08*** |
| Rammasun | 23 | 1.33 | 3 | 137.51*** | 14823.2*** |
| Rammasun | 24 | 1.32 | 3 | 160.55*** | 14108.83*** |
| Rammasun | 25 | 1.32 | 3 | 168.09*** | 14820.26*** |
| Rammasun | 26 | 1.32 | 3 | 181.81*** | 13917.71*** |
| Rammasun | 27 | 1.38 | 3 | 153.75*** | 17093.03*** |
| Rammasun | 28 | 1.43 | 3 | 106.01*** | 17565.41*** |
| Rammasun | 29 | 1.37 | 3 | 140.13*** | 14694.57*** |
| Rammasun | 30 | 1.43 | 3 | 111.61*** | 17549.01*** |
| Rammasun | 31 | 1.33 | 3 | 152.28*** | 14782.05*** |
| Rammasun | 32 | 1.33 | 3 | 140.47*** | 15427.2*** |
| Rammasun | 33 | 1.32 | 3 | 162.89*** | 15268.24*** |
| Rammasun | 34 | 1.41 | 3 | 138.03*** | 18930.48*** |
| Kalmaegi | 1 | 1.25 | 1 | 15.09*** | 1358.09*** |
| Kalmaegi | 2 | 1.23 | 1 | 19.81*** | 1360.07*** |
| Kalmaegi | 3 | 1.26 | 1 | 15.49*** | 1881.58*** |
| Kalmaegi | 4 | 1.21 | 1 | 16.2*** | 1210.52*** |
| Kalmaegi | 5 | 1.28 | 1 | 9.47*** | 1364.42*** |
| Kalmaegi | 6 | 1.16 | 1 | 22.79*** | 1073.37*** |
| Kalmaegi | 7 | 1.35 | 1 | 5.97** | 1919.13*** |
| Kalmaegi | 8 | 1.24 | 1 | 11.89*** | 1188.82*** |
| Kalmaegi | 9 | 1.41 | 1 | 5.3** | 1935.71*** |
| Kalmaegi | 10 | 1.20 | 1 | 18.7*** | 1061.85*** |
| Kalmaegi | 11 | 1.24 | 1 | 7.99*** | 746.2*** |
| Kalmaegi | 12 | 1.21 | 1 | 11.2*** | 722.52*** |
| Kalmaegi | 13 | 1.31 | 1 | 4.54* | 813.5*** |
| Kalmaegi | 14 | 1.10 | 1 | 12.39*** | 420.08*** |
| Kalmaegi | 15 | 1.13 | 1 | 23.15*** | 825.2*** |
| Kalmaegi | 16 | 1.40 | 1 | -1 | 1852.92*** |
| Kalmaegi | 17 | 1.28 | 1 | 17.05*** | 2389.73*** |
| Kalmaegi | 18 | 1.30 | 1 | 8.82** | 1721.6*** |
| Kalmaegi | 19 | 1.23 | 1 | 18.26*** | 1341.14*** |
| Kalmaegi | 20 | 1.28 | 1 | 9.86*** | 1533.66*** |
| Kalmaegi | 21 | 1.20 | 1 | 17.74*** | 975.47*** |
| Kalmaegi | 22 | 1.22 | 1 | 15.3*** | 1298.1*** |
| Kalmaegi | 23 | 1.29 | 1 | 19.05*** | 1962.5*** |
| Kalmaegi | 24 | 1.22 | 1 | 32.6*** | 2454.46*** |
| Kalmaegi | 25 | 1.27 | 1 | 13.47** | 1465.49*** |
| Kalmaegi | 26 | 1.28 | 1 | 5.73** | 838.39*** |
| Kalmaegi | 27 | 1.15 | 1 | 16.47*** | 630.19*** |
| Kalmaegi | 28 | 1.13 | 1 | 19.64*** | 620.27*** |
| Kalmaegi | 29 | 1.16 | 1 | 28.82*** | 1091.75*** |
| Kalmaegi | 30 | 1.25 | 1 | 12.46** | 1736.08*** |
| Kalmaegi | 31 | 1.13 | 1 | 41.48*** | 1296.58*** |
| Kalmaegi | 32 | 1.25 | 1 | 8.37** | 904.67*** |
| Kalmaegi | 33 | 1.16 | 1 | 21.63*** | 848.72*** |
| Kalmaegi | 34 | 1.19 | 1 | 17.22*** | 875.95*** |
| Bohol | 1 | 1.12 | 1 | 71.11*** | 3544.69*** |
| Bohol | 2 | 1.08 | 1 | 75.93*** | 2719.24*** |
| Bohol | 3 | 1.11 | 1 | 60.23*** | 2896.21*** |
| Bohol | 4 | 1.08 | 1 | 72.04*** | 2910.26*** |
| Bohol | 5 | 1.09 | 1 | 81.69*** | 3377.03*** |
| Bohol | 6 | 1.10 | 1 | 104.64*** | 3978.45*** |
| Bohol | 7 | 1.15 | 1 | 82.04*** | 4438.49*** |
| Bohol | 8 | 1.10 | 1 | 68.9*** | 3046.07*** |
| Bohol | 9 | 1.11 | 1 | 53.44*** | 3563.82*** |
| Bohol | 10 | 1.09 | 1 | 67.34*** | 2745.31*** |
| Bohol | 11 | 1.10 | 1 | 71.39*** | 3494.55*** |
| Bohol | 12 | 1.07 | 1 | 83.1*** | 2892.98*** |
| Bohol | 13 | 1.11 | 1 | 76.68*** | 3981.62*** |
| Bohol | 14 | 1.16 | 1 | 74.21*** | 5168.08*** |
| Bohol | 15 | 1.13 | 1 | 57.04*** | 2954.24*** |
| Bohol | 16 | 1.21 | 1 | 87.28*** | 12052.22*** |
| Bohol | 17 | 1.21 | 1 | 54.65*** | 8646.87*** |
| Bohol | 18 | 1.22 | 1 | 51.86*** | 6694.7*** |
| Bohol | 19 | 1.19 | 1 | 65.68*** | 5373.59*** |
| Bohol | 20 | 1.14 | 1 | 71.3*** | 4608.66*** |
| Bohol | 21 | 1.20 | 1 | 61.73*** | 8685.52*** |
| Bohol | 22 | 1.23 | 1 | 44.04*** | 6676.7*** |
| Bohol | 23 | 1.23 | 1 | 46.88*** | 5834.97*** |
| Bohol | 24 | 1.22 | 1 | 44.53*** | 5231.74*** |
| Bohol | 25 | 1.19 | 1 | 46.19*** | 4857.74*** |
| Bohol | 26 | 1.18 | 1 | 61.75*** | 4705.8*** |
| Bohol | 27 | 1.16 | 1 | 67.2*** | 4103.95*** |
| Bohol | 28 | 1.22 | 1 | 42.97*** | 4922.36*** |
| Bohol | 29 | 1.22 | 1 | 44.02*** | 6122.71*** |
| Bohol | 30 | 1.21 | 1 | 47.64*** | 4787.69*** |
| Bohol | 31 | 1.21 | 1 | 37.65*** | 5647.93*** |
| Iquique | 1 | 1.43 | 3 | 0.56 | 216.18*** |
| Iquique | 2 | 1.30 | 3 | 3.48* | 462.83*** |
| Iquique | 3 | 1.32 | 3 | 3.66** | 346.26*** |
| Iquique | 4 | 1.21 | 3 | 0.15 | 309.43*** |
| Iquique | 5 | 1.18 | 3 | -0.56 | 409.82*** |
| Iquique | 6 | 1.28 | 3 | -2.02 | 345.18*** |
| Iquique | 7 | 1.32 | 3 | -9.04* | 459.31*** |
| Iquique | 8 | 1.20 | 3 | 5.7* | 547.9*** |
| Iquique | 9 | 1.16 | 3 | 8.5*** | 284.93*** |
| Iquique | 10 | 1.11 | 3 | 9.16*** | 220.76*** |
| Iquique | 11 | 1.16 | 3 | 5.95*** | 174.12*** |
| Iquique | 12 | 1.07 | 3 | 9.35*** | 187.97*** |
| Iquique | 13 | 1.24 | 3 | 2.41 | 255.43*** |
| Iquique | 14 | 1.37 | 3 | 0.73 | 330.84*** |
| Iquique | 15 | 1.15 | 3 | 8.33*** | 337.83*** |
| Iquique | 16 | 1.07 | 3 | 6.61** | 204.82*** |
| Iquique | 17 | 1.12 | 3 | -4.76 | 428.9*** |
| Iquique | 18 | 1.23 | 3 | 6.5*** | 535.66*** |
| Iquique | 19 | 1.32 | 3 | 5.02*** | 976.68*** |
| Iquique | 20 | 1.31 | 3 | 5.39*** | 540.19*** |
| Iquique | 21 | 1.26 | 3 | 5.39*** | 460.8*** |
| Iquique | 22 | 1.25 | 3 | 6.24*** | 402.64*** |
| Iquique | 23 | 1.36 | 3 | 2.61* | 394.95*** |
| Iquique | 24 | 1.32 | 3 | 3.74** | 628.69*** |
| Iquique | 25 | 1.32 | 3 | 4.05** | 522.83*** |
| Iquique | 26 | 1.35 | 3 | 2.58** | 488.06*** |
| Iquique | 27 | 1.34 | 3 | -4.71 | 445.09*** |
| Iquique | 28 | 1.43 | 3 | 1.46 | 538.78*** |
| Iquique | 29 | 1.35 | 3 | 6.46*** | 833.31*** |
| Iquique | 30 | 1.28 | 3 | 12.27*** | 1369.89*** |
| Iquique | 31 | 1.35 | 3 | 10.22*** | 1410.85*** |
| Napa | 1 | 1.42 | 6 | 7.15*** | 1272.13*** |
| Napa | 2 | 1.47 | 6 | 5.83*** | 1038.89*** |
| Napa | 3 | 1.38 | 6 | 11.81*** | 1118.2*** |
| Napa | 4 | 1.34 | 6 | 15.61*** | 1054.13*** |
| Napa | 5 | 1.49 | 6 | 6.99*** | 1539.98*** |
| Napa | 6 | 1.43 | 6 | 8.56*** | 1119.93*** |
| Napa | 7 | 1.41 | 6 | 11.46*** | 1266.87*** |
| Napa | 8 | 1.49 | 6 | 6.78*** | 1280.35*** |
| Napa | 9 | 1.49 | 6 | 6.62*** | 1457.53*** |
| Napa | 10 | 1.39 | 6 | 9.58*** | 1111.43*** |
| Napa | 11 | 1.35 | 6 | 13.28*** | 896.88*** |
| Napa | 12 | 1.47 | 6 | 6.53*** | 1088.76*** |
| Napa | 13 | 1.44 | 6 | 8.13*** | 1243.46*** |
| Napa | 14 | 1.47 | 6 | 7.29*** | 1252.18*** |
| Napa | 15 | 1.37 | 6 | 16.05*** | 1149.79*** |
| Napa | 16 | 1.38 | 6 | 13.69*** | 1018.2*** |
| Napa | 17 | 1.33 | 6 | 19.2*** | 1050.78*** |
| Napa | 18 | 1.35 | 6 | 13.45*** | 970.62*** |
| Napa | 19 | 1.45 | 6 | 6.86*** | 1026.65*** |
| Napa | 20 | 1.38 | 6 | 11.79*** | 1107.22*** |
| Napa | 21 | 1.36 | 6 | 13.8*** | 980.09*** |
| Napa | 22 | 1.38 | 6 | 14.52*** | 1190.63*** |
| Napa | 23 | 1.34 | 6 | 16.61*** | 1058.66*** |
| Napa | 24 | 1.28 | 6 | 21.57*** | 873.68*** |
| Napa | 25 | 1.43 | 6 | 9.94*** | 1376.51*** |
| Napa | 26 | 1.23 | 6 | 65.3*** | 2241.0*** |
| Napa | 27 | 1.38 | 6 | 18.88*** | 1485.56*** |
| Napa | 28 | 1.42 | 6 | 16.3*** | 1690.35*** |
| Napa | 29 | 1.35 | 6 | 16.17*** | 989.29*** |
| Napa | 30 | 1.32 | 6 | 19.14*** | 949.33*** |
| Napa | 31 | 1.28 | 6 | 19.36*** | 848.0*** |
| Norfolk | 1 | 1.30 | 3 | 41.95*** | 5292.83*** |
| Norfolk | 2 | 1.31 | 3 | 40.04*** | 4960.98*** |
| Norfolk | 3 | 1.28 | 3 | 36.43*** | 3958.71*** |
| Norfolk | 4 | 1.35 | 3 | 27.48*** | 5166.28*** |
| Norfolk | 5 | 1.37 | 3 | 21.29*** | 5388.42*** |
| Norfolk | 6 | 1.29 | 3 | 41.26*** | 4524.78*** |
| Norfolk | 7 | 1.28 | 3 | 40.61*** | 4498.48*** |
| Norfolk | 8 | 1.30 | 3 | 33.79*** | 4280.03*** |
| Norfolk | 9 | 1.30 | 3 | 33.17*** | 3991.5*** |
| Norfolk | 10 | 1.23 | 3 | 54.12*** | 3775.52*** |
| Norfolk | 11 | 1.30 | 3 | 26.63*** | 4055.89*** |
| Norfolk | 12 | 1.37 | 3 | 20.7*** | 5284.65*** |
| Norfolk | 13 | 1.28 | 3 | 38.35*** | 4076.61*** |
| Norfolk | 14 | 1.32 | 3 | 30.54*** | 4376.95*** |
| Norfolk | 15 | 1.31 | 3 | 36.82*** | 4905.99*** |
| Norfolk | 16 | 1.30 | 3 | 39.55*** | 5049.72*** |
| Norfolk | 17 | 1.29 | 3 | 36.3*** | 4299.86*** |
| Norfolk | 18 | 1.32 | 3 | 30.72*** | 4363.66*** |
| Norfolk | 19 | 1.40 | 3 | 14.13*** | 5444.36*** |
| Norfolk | 20 | 1.28 | 3 | 39.54*** | 4495.37*** |
| Norfolk | 21 | 1.28 | 3 | 39.19*** | 4184.33*** |
| Norfolk | 22 | 1.30 | 3 | 31.86*** | 4333.82*** |
| Norfolk | 23 | 1.29 | 3 | 35.5*** | 4203.18*** |
| Norfolk | 24 | 1.27 | 3 | 40.11*** | 4376.94*** |
| Norfolk | 25 | 1.35 | 3 | 25.43*** | 5344.27*** |
| Norfolk | 26 | 1.42 | 3 | 13.53*** | 6591.65*** |
| Norfolk | 27 | 1.33 | 3 | 26.8*** | 4825.96*** |
| Norfolk | 28 | 1.35 | 3 | 21.51*** | 4851.11*** |
| Norfolk | 29 | 1.32 | 3 | 28.36*** | 4399.09*** |
| Norfolk | 30 | 1.31 | 3 | 31.18*** | 4600.62*** |
| Norfolk | 31 | 1.29 | 3 | 39.69*** | 4575.32*** |
| Hamburg | 1 | 1.12 | 1 | 16.42*** | 621.79*** |
| Hamburg | 2 | 1.05 | 1 | 17.73*** | 453.31*** |
| Hamburg | 3 | 1.05 | 1 | 11.89*** | 404.06*** |
| Hamburg | 4 | 1.11 | 1 | 5.89* | 395.68*** |
| Hamburg | 5 | 1.12 | 1 | 4.76 | 488.16*** |
| Hamburg | 6 | 1.06 | 1 | 13.29*** | 510.73*** |
| Hamburg | 7 | 1.04 | 1 | 13.17*** | 509.95*** |
| Hamburg | 8 | 1.02 | 1 | 18.81*** | 389.85*** |
| Hamburg | 9 | 1.03 | 1 | 9.19** | 359.7*** |
| Hamburg | 10 | 1.00 | 1 | 17.22*** | 426.04*** |
| Hamburg | 11 | 1.07 | 1 | 6.53* | 475.63*** |
| Hamburg | 12 | 1.07 | 1 | 5.29* | 324.77*** |
| Hamburg | 13 | 1.00 | 1 | 13.85*** | 206.52*** |
| Hamburg | 14 | 1.00 | 1 | 11.87*** | 355.13*** |
| Hamburg | 15 | 1.01 | 1 | 15.7*** | 371.85*** |
| Hamburg | 16 | 1.11 | 1 | 1.37 | 658.46*** |
| Hamburg | 17 | 1.06 | 1 | 10.12** | 500.3*** |
| Hamburg | 18 | 1.06 | 1 | 5.54 | 392.94*** |
| Hamburg | 19 | 1.16 | 1 | 3.32 | 384.85*** |
| Hamburg | 20 | 1.06 | 1 | 11.66*** | 387.39*** |
| Hamburg | 21 | 1.00 | 1 | 16.05*** | 223.67*** |
| Hamburg | 22 | 1.01 | 1 | 12.83*** | 350.58*** |
| Hamburg | 23 | 1.07 | 1 | 12.95*** | 456.78*** |
| Hamburg | 24 | 1.00 | 1 | 20.39*** | 296.3*** |
| Hamburg | 25 | 1.01 | 1 | 11.44*** | 295.37*** |
| Hamburg | 26 | 1.07 | 1 | 6.32** | 278.46*** |
| Hamburg | 27 | 1.00 | 1 | 22.29*** | 224.28*** |
| Hamburg | 28 | 1.00 | 1 | 17.06*** | 205.88*** |
| Hamburg | 29 | 1.00 | 1 | 18.0*** | 307.77*** |
| Hamburg | 30 | 1.04 | 1 | 10.35*** | 301.97*** |
| Hamburg | 31 | 1.00 | 1 | 17.94*** | 332.82*** |
| Atlanta | 1 | 1.29 | 5 | 47.51*** | 3912.13*** |
| Atlanta | 2 | 1.30 | 5 | 48.89*** | 3706.45*** |
| Atlanta | 3 | 1.26 | 5 | 62.1*** | 3604.21*** |
| Atlanta | 4 | 1.29 | 5 | 56.81*** | 3686.4*** |
| Atlanta | 5 | 1.37 | 5 | 31.23*** | 4042.66*** |
| Atlanta | 6 | 1.32 | 5 | 49.09*** | 3710.96*** |
| Atlanta | 7 | 1.32 | 5 | 43.36*** | 4029.07*** |
| Atlanta | 8 | 1.31 | 5 | 51.45*** | 4202.53*** |
| Atlanta | 9 | 1.33 | 5 | 35.54*** | 3929.05*** |
| Atlanta | 10 | 1.27 | 5 | 63.22*** | 4273.64*** |
| Atlanta | 11 | 1.28 | 5 | 63.96*** | 4381.42*** |
| Atlanta | 12 | 1.25 | 5 | 74.01*** | 4484.44*** |
| Atlanta | 13 | 1.39 | 5 | 28.19*** | 5187.56*** |
| Atlanta | 14 | 1.28 | 5 | 60.27*** | 4281.44*** |
| Atlanta | 15 | 1.30 | 5 | 76.06*** | 6736.03*** |
| Atlanta | 16 | 1.45 | 5 | -22.6** | 5395.63*** |
| Atlanta | 17 | 1.43 | 5 | 26.24*** | 5538.64*** |
| Atlanta | 18 | 1.30 | 5 | 61.89*** | 4674.06*** |
| Atlanta | 19 | 1.27 | 5 | 74.23*** | 4608.4*** |
| Atlanta | 20 | 1.38 | 5 | 33.99*** | 5263.23*** |
| Atlanta | 21 | 1.34 | 5 | 44.02*** | 4575.93*** |
| Atlanta | 22 | 1.31 | 5 | 60.59*** | 4791.65*** |
| Atlanta | 23 | 1.30 | 5 | 53.82*** | 4041.89*** |
| Atlanta | 24 | 1.24 | 5 | 72.12*** | 3582.09*** |
| Atlanta | 25 | 1.23 | 5 | 82.96*** | 4097.88*** |
| Atlanta | 26 | 1.25 | 5 | 65.32*** | 4061.39*** |
| Atlanta | 27 | 1.36 | 5 | 36.14*** | 4929.58*** |
| Atlanta | 28 | 1.26 | 5 | 72.13*** | 4738.58*** |
| Atlanta | 29 | 1.42 | 5 | 22.51*** | 5702.91*** |
| Atlanta | 30 | 1.54 | 5 | -19.62** | 6075.7*** |
| Atlanta | 31 | 1.45 | 5 | 11.82*** | 5463.94*** |
| Phoenix | 1 | 1.37 | 5 | 153.65*** | 25988.16*** |
| Phoenix | 2 | 1.28 | 5 | 309.86*** | 22205.19*** |
| Phoenix | 3 | 1.28 | 5 | 339.11*** | 23940.53*** |
| Phoenix | 4 | 1.24 | 5 | 349.67*** | 20177.3*** |
| Phoenix | 5 | 1.23 | 5 | 406.02*** | 21228.94*** |
| Phoenix | 6 | 1.21 | 5 | 437.94*** | 19006.33*** |
| Phoenix | 7 | 1.31 | 5 | 241.63*** | 23106.08*** |
| Phoenix | 8 | 1.34 | 5 | 212.37*** | 26660.57*** |
| Phoenix | 9 | 1.41 | 5 | 123.89*** | 28031.78*** |
| Phoenix | 10 | 1.30 | 5 | 308.21*** | 26497.92*** |
| Phoenix | 11 | 1.29 | 5 | 317.96*** | 23911.48*** |
| Phoenix | 12 | 1.29 | 5 | 327.89*** | 25244.33*** |
| Phoenix | 13 | 1.21 | 5 | 439.64*** | 19173.58*** |
| Phoenix | 14 | 1.30 | 5 | 270.64*** | 22604.58*** |
| Phoenix | 15 | 1.42 | 5 | 122.23*** | 29565.39*** |
| Phoenix | 16 | 1.34 | 5 | 326.38*** | 36424.54*** |
| Phoenix | 17 | 1.30 | 5 | 301.39*** | 23966.43*** |
| Phoenix | 18 | 1.27 | 5 | 335.89*** | 22357.02*** |
| Phoenix | 19 | 1.26 | 5 | 341.7*** | 21486.78*** |
| Phoenix | 20 | 1.19 | 5 | 463.78*** | 17837.27*** |
| Phoenix | 21 | 1.29 | 5 | 275.61*** | 21919.64*** |
| Phoenix | 22 | 1.40 | 5 | 145.52*** | 28327.03*** |
| Phoenix | 23 | 1.29 | 5 | 315.77*** | 23951.23*** |
| Phoenix | 24 | 1.23 | 5 | 445.82*** | 25741.24*** |
| Phoenix | 25 | 1.25 | 5 | 315.34*** | 18815.52*** |
| Phoenix | 26 | 1.23 | 5 | 268.62*** | 13802.69*** |
| Phoenix | 27 | 1.17 | 5 | 339.66*** | 12313.97*** |
| Phoenix | 28 | 1.27 | 5 | 208.94*** | 14869.42*** |
| Phoenix | 29 | 1.36 | 5 | 132.07*** | 18912.31*** |
| Phoenix | 30 | 1.29 | 5 | 200.42*** | 15388.26*** |
| Phoenix | 31 | 1.27 | 5 | 221.81*** | 15441.31*** |
| Detroit | 1 | 1.37 | 5 | 307.41*** | 36677.93*** |
| Detroit | 2 | 1.36 | 5 | 279.44*** | 31541.65*** |
| Detroit | 3 | 1.33 | 5 | 323.61*** | 31137.64*** |
| Detroit | 4 | 1.34 | 5 | 312.75*** | 31350.02*** |
| Detroit | 5 | 1.29 | 5 | 361.37*** | 27063.4*** |
| Detroit | 6 | 1.24 | 5 | 510.72*** | 30125.61*** |
| Detroit | 7 | 1.31 | 5 | 379.34*** | 34327.56*** |
| Detroit | 8 | 1.38 | 5 | 215.45*** | 32300.68*** |
| Detroit | 9 | 1.39 | 5 | 235.14*** | 34447.59*** |
| Detroit | 10 | 1.38 | 5 | 254.73*** | 31710.3*** |
| Detroit | 11 | 1.33 | 5 | 349.79*** | 31395.0*** |
| Detroit | 12 | 1.30 | 5 | 409.6*** | 30399.96*** |
| Detroit | 13 | 1.27 | 5 | 419.26*** | 27414.83*** |
| Detroit | 14 | 1.40 | 5 | 204.05*** | 32676.09*** |
| Detroit | 15 | 1.38 | 5 | 234.61*** | 30693.32*** |
| Detroit | 16 | 1.35 | 5 | 269.08*** | 29431.99*** |
| Detroit | 17 | 1.34 | 5 | 318.81*** | 29393.67*** |
| Detroit | 18 | 1.32 | 5 | 354.77*** | 31516.87*** |
| Detroit | 19 | 1.30 | 5 | 356.54*** | 27722.4*** |
| Detroit | 20 | 1.29 | 5 | 357.94*** | 26077.83*** |
| Detroit | 21 | 1.33 | 5 | 266.64*** | 25613.08*** |
| Detroit | 22 | 1.39 | 5 | 108.64*** | 15689.28*** |
| Detroit | 23 | 1.32 | 5 | 159.81*** | 14285.39*** |
| Detroit | 24 | 1.30 | 5 | 168.76*** | 12445.07*** |
| Detroit | 25 | 1.28 | 5 | 191.85*** | 12479.36*** |
| Detroit | 26 | 1.24 | 5 | 222.52*** | 11473.54*** |
| Detroit | 27 | 1.29 | 5 | 161.17*** | 11317.58*** |
| Detroit | 28 | 1.34 | 5 | 119.71*** | 12266.16*** |
| Detroit | 29 | 1.30 | 5 | 180.34*** | 13461.11*** |
| Detroit | 30 | 1.31 | 5 | 145.59*** | 12267.05*** |
| Detroit | 31 | 1.34 | 5 | 162.21*** | 14211.81*** |
| Baltimore | 1 | 1.34 | 5 | 120.86*** | 9411.18*** |
| Baltimore | 2 | 1.35 | 5 | 103.34*** | 9793.81*** |
| Baltimore | 3 | 1.36 | 5 | 107.42*** | 10306.4*** |
| Baltimore | 4 | 1.34 | 5 | 121.75*** | 9723.51*** |
| Baltimore | 5 | 1.31 | 5 | 144.88*** | 9963.87*** |
| Baltimore | 6 | 1.29 | 5 | 112.26*** | 8728.37*** |
| Baltimore | 7 | 1.38 | 5 | 91.37*** | 10223.88*** |
| Baltimore | 8 | 1.36 | 5 | 101.02*** | 9747.42*** |
| Baltimore | 9 | 1.32 | 5 | 108.61*** | 8556.41*** |
| Baltimore | 10 | 1.36 | 5 | 112.14*** | 10365.85*** |
| Baltimore | 11 | 1.33 | 5 | 133.76*** | 10430.14*** |
| Baltimore | 12 | 1.27 | 5 | 177.03*** | 10137.35*** |
| Baltimore | 13 | 1.27 | 5 | 149.61*** | 9112.38*** |
| Baltimore | 14 | 1.32 | 5 | 113.53*** | 9856.63*** |
| Baltimore | 15 | 1.35 | 5 | 109.67*** | 10673.02*** |
| Baltimore | 16 | 1.42 | 5 | 82.13*** | 12484.44*** |
| Baltimore | 17 | 1.34 | 5 | 117.45*** | 10083.22*** |
| Baltimore | 18 | 1.35 | 5 | 107.56*** | 9287.11*** |
| Baltimore | 19 | 1.29 | 5 | 129.47*** | 8353.46*** |
| Baltimore | 20 | 1.32 | 5 | 125.04*** | 8700.17*** |
| Baltimore | 21 | 1.42 | 5 | 69.1*** | 10340.08*** |
| Baltimore | 22 | 1.40 | 5 | 89.44*** | 11581.29*** |
| Baltimore | 23 | 1.40 | 5 | 77.54*** | 11956.29*** |
| Baltimore | 24 | 1.37 | 5 | 111.48*** | 11455.12*** |
| Baltimore | 25 | 1.32 | 5 | 118.46*** | 8854.2*** |
| Baltimore | 26 | 1.34 | 5 | 114.61*** | 9238.23*** |
| Baltimore | 27 | 1.36 | 5 | 92.9*** | 8990.97*** |
| Baltimore | 28 | 1.41 | 5 | 74.74*** | 10126.66*** |
| Baltimore | 29 | 1.33 | 5 | 129.08*** | 9752.21*** |
| Baltimore | 30 | 1.31 | 5 | 135.56*** | 9439.43*** |
| Baltimore | 31 | 1.24 | 5 | 166.69*** | 7244.04*** |
| AuFire1 | 1 | 1.38 | 5 | 8.32*** | 1128.1*** |
| AuFire1 | 2 | 1.36 | 5 | 7.22*** | 1126.33*** |
| AuFire1 | 3 | 1.28 | 5 | 15.99*** | 1425.95*** |
| AuFire1 | 4 | 1.34 | 5 | 8.5*** | 1271.22*** |
| AuFire1 | 5 | 1.30 | 5 | 13.3*** | 1398.02*** |
| AuFire1 | 6 | 1.42 | 5 | 4.51*** | 1250.05*** |
| AuFire1 | 7 | 1.34 | 5 | 6.79*** | 824.3*** |
| AuFire1 | 8 | 1.34 | 5 | 7.6*** | 996.69*** |
| AuFire1 | 9 | 1.29 | 5 | 7.97*** | 993.17*** |
| AuFire1 | 10 | 1.29 | 5 | 15.17*** | 1480.55*** |
| AuFire1 | 11 | 1.26 | 5 | 12.68*** | 1046.9*** |
| AuFire1 | 12 | 1.31 | 5 | 7.29*** | 742.98*** |
| AuFire1 | 13 | 1.33 | 5 | 6.75*** | 846.97*** |
| AuFire1 | 14 | 1.39 | 5 | 6.42*** | 1312.8*** |
| AuFire1 | 15 | 1.28 | 5 | 7.07*** | 770.35*** |
| AuFire1 | 16 | 1.25 | 5 | 11.53*** | 995.34*** |
| AuFire1 | 17 | 1.23 | 5 | 13.5*** | 782.51*** |
| AuFire1 | 18 | 1.24 | 5 | 13.23*** | 864.82*** |
| AuFire1 | 19 | 1.35 | 5 | 7.82*** | 1395.08*** |
| AuFire1 | 20 | 1.45 | 5 | 5.93*** | 1758.1*** |
| AuFire1 | 21 | 1.36 | 5 | 7.88*** | 1488.44*** |
| AuFire1 | 22 | 1.37 | 5 | 7.01*** | 1362.07*** |
| AuFire1 | 23 | 1.35 | 5 | 8.51*** | 1502.27*** |
| AuFire1 | 24 | 1.29 | 5 | 14.75*** | 1313.13*** |
| AuFire1 | 25 | 1.41 | 5 | 6.95*** | 1605.14*** |
| AuFire1 | 26 | 1.39 | 5 | 1.48 | 1368.14*** |
| AuFire1 | 27 | 1.34 | 5 | 10.04*** | 1151.24*** |
| AuFire1 | 28 | 1.33 | 5 | 10.93*** | 1269.62*** |
| AuFire2 | 1 | 1.35 | 5 | 19.77*** | 2412.84*** |
| AuFire2 | 2 | 1.25 | 5 | 28.99*** | 2312.84*** |
| AuFire2 | 3 | 1.26 | 5 | 34.63*** | 2446.69*** |
| AuFire2 | 4 | 1.24 | 5 | 33.47*** | 2146.04*** |
| AuFire2 | 5 | 1.21 | 5 | 29.11*** | 1693.35*** |
| AuFire2 | 6 | 1.34 | 5 | 14.29*** | 2026.56*** |
| AuFire2 | 7 | 1.25 | 5 | 27.37*** | 1702.49*** |
| AuFire2 | 8 | 1.24 | 5 | 29.71*** | 1656.73*** |
| AuFire2 | 9 | 1.23 | 5 | 33.11*** | 1640.66*** |
| AuFire2 | 10 | 1.21 | 5 | 45.57*** | 2165.76*** |
| AuFire2 | 11 | 1.21 | 5 | 35.37*** | 1752.44*** |
| AuFire2 | 12 | 1.20 | 5 | 32.07*** | 1506.98*** |
| AuFire2 | 13 | 1.31 | 5 | 21.51*** | 1819.72*** |
| AuFire2 | 14 | 1.31 | 5 | 25.51*** | 2549.98*** |
| AuFire2 | 15 | 1.27 | 5 | 28.06*** | 2000.29*** |
| AuFire2 | 16 | 1.27 | 5 | 32.64*** | 2263.94*** |
| AuFire2 | 17 | 1.21 | 5 | 35.68*** | 1809.06*** |
| AuFire2 | 18 | 1.22 | 5 | 38.94*** | 1807.62*** |
| AuFire2 | 19 | 1.29 | 5 | 31.14*** | 2437.33*** |
| AuFire2 | 20 | 1.39 | 5 | 15.12*** | 2918.18*** |
| AuFire2 | 21 | 1.31 | 5 | 24.01*** | 2338.01*** |
| AuFire2 | 22 | 1.27 | 5 | 27.53*** | 1867.91*** |
| AuFire2 | 23 | 1.26 | 5 | 31.47*** | 2155.15*** |
| AuFire2 | 24 | 1.22 | 5 | 41.03*** | 2040.38*** |
| AuFire2 | 25 | 1.33 | 5 | 22.44*** | 2546.69*** |
| AuFire2 | 26 | 1.32 | 5 | 19.84*** | 2294.59*** |
| AuFire2 | 27 | 1.26 | 5 | 35.66*** | 2172.5*** |
| AuFire2 | 28 | 1.30 | 5 | 25.89*** | 2438.27*** |

^1^ Xmin is the minimum distance that was used to fit to the truncated power-law distribution

^2^ Positive value means the fitting favored truncated power-law. Negative means the fitting favored lognormal distribution

^3^ Positive value means the fitting favored truncated power-law. Negative means the fitting favored exponential distribution

*significant at *p*< 0.05; ** significant at *p*<0.01; ***significant at *p*<0.001
